# Off-pump coronary artery bypass grafting versus optimal medical therapy: effectiveness of incomplete surgical myocardial revascularization in high-risk patients with multi-vessel coronary artery disease

**DOI:** 10.1186/1749-8090-10-S1-A351

**Published:** 2015-12-16

**Authors:** Cristiano Spadaccio, Antonio Nenna, Filippo Prestipino, Gwyn W Beattie, Francesco Nappi, Massimo Chello, Fraser W Sutherland

**Affiliations:** 1 Department of Cardiothoracic Surgery, Golden Jubilee National Hospital, Glasgow, G814DY, UK; 2 Department of Cardiac Surgery University Campus Bio-Medico of Rome, Rome, Italy; 3 Cardiac Surgery, Centre Cardiologique du Nord de Saint-Denis, Paris, France

## Background/Introduction

High-risk patients with multivessel disease (MVD) represent a surgical challenge carrying high mortality risk. These cases elicit discussion within heart teams regarding the actual benefit of undertaking major surgery on these patients and often lead to abandon the surgical option. Off-pump coronary artery bypass (OPCAB) provides good quality graft on left anterior descending (LAD) without exposing the patient to cardiopulmonary bypass, and, despite providing an incomplete revascularization, might be the ideal choice in patients with multiple comorbidities, not eligible to percutaneous or on-pump procedures.

## Aims/Objectives

To compare survival in high-risk patients with MVD and no percutaneous option, treated with incomplete off-pump surgical myocardial revascularization or discharged on optimal medical therapy.

## Method

83 high-risk patients with MVD were enrolled: 42 were treated with incomplete off-pump revascularization using left internal mammary artery (LIMA) to LAD; 41 were treated with optimal medical therapy (OMT), having refused surgery. Patients were followed-up by telephone interview. Primary endpoint was survival from all-cause mortality; secondary endpoints were survival from cardiac-related mortality and freedom from non-fatal major adverse cardiac events (MACE).

## Results

During follow up, there were 11 deaths in OPCAB group and 27 deaths in OMT group. Death was due to cardiac factors in 6 and 15 patients, respectively. MACEs were observed in 6 patients in OPCAB group and 4 patients in OMT group. Both survival from all-cause mortality and cardiac-related events were in favor of the OPCAB group over the OMT which carried a propensity score-adjusted hazard ratio of 3.862 and 3.663, for all-cause and cardiac-related mortality respectively. There was no statistically significant difference concerning freedom from MACE.

## Discussion/Conclusion

For high-risk patients with MVD, considered ineligible for on-pump complete revascularization surgery or percutaneous coronary intervention, incomplete revascularization with OPCAB LIMA-on-LAD offers benefits in survival when compared to OMT alone.

